# 1016. A Qualitative Study of Patient Perspectives on the Use of Antibiotics Without a Prescription

**DOI:** 10.1093/ofid/ofad500.047

**Published:** 2023-11-27

**Authors:** Lindsey Laytner, Patricia Chen, Susan G Nash, Juanita Salinas, Kiara Olmeda, Roger Zoorob, Michael Paasche-Orlow, Barbara Trautner, Larissa Grigoryan

**Affiliations:** Baylor College of Medicine, Department of Family and Community Medicine, Houston, TX; Baylor College of Medicine, Houston, Texas; Baylor College of Medicine, Houston, Texas; Baylor College of Medicine, Houston, Texas; Baylor College of Medicine, Houston, Texas; Baylor College of Medicine, Houston, Texas; Tufts University, Boston, Massachusetts; Michael E. DeBakey Veterans Affairs Medical Center / Baylor College of Medicine, Houston, TX; Baylor College of Medicine, Houston, Texas

## Abstract

**Background:**

Using antibiotics without medical guidance (nonprescription use) can be unsafe and increase antimicrobial resistance rates. Few studies have qualitatively examined patient perspectives of nonprescription use in the US. To understand the reasons and motivations underlying patients’ nonprescription use, we explored the perceptions and experiences of sociodemographically diverse patients served by two US healthcare systems.

**Methods:**

We conducted semi-structured interviews with outpatients who had endorsed nonprescription use on a previous survey in public and private healthcare systems in Texas. Interviews were conducted remotely in English or Spanish from May 2020 through October 2021. We used inductive coding and the Kilbourne framework for health disparities research to guide our thematic analysis.

**Results:**

Overall, 86 participants completed interviews. Over 70% of participants were female, Hispanic or African American, educated (high school or above), and attended publicly-funded clinics (Table 1). In-depth interviews revealed seven themes (reasons/motives) driving nonprescription use (Figure 1). Participants reported using nonprescription antibiotics to relieve respiratory symptoms or pain, bypass healthcare-access barriers, and for convenience. Participants stated that they “know their bodies” when sick and expressed that antibiotics are “like gold” (valuable/effective) and can be used when over-the-counter medicines do not work and when the antibiotics or source of the antibiotics are “trusted.” Table 2 highlights representative participant quotes by theme.

**Table 1.**
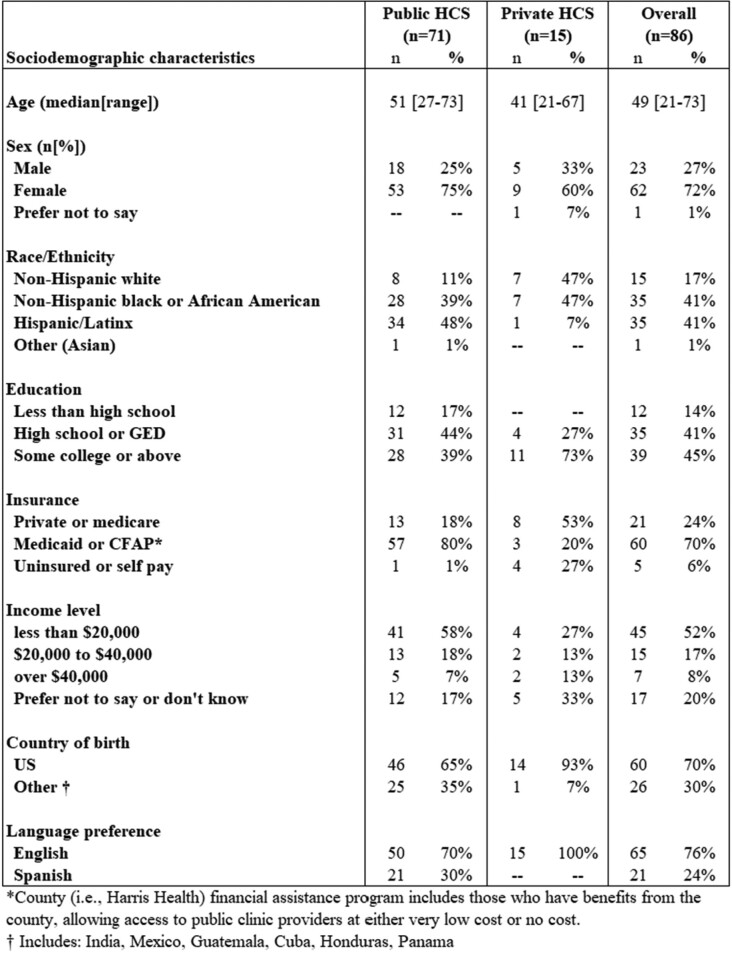
Sociodemographic characteristics of participants by healthcare system (HCS)

**Table 2.**
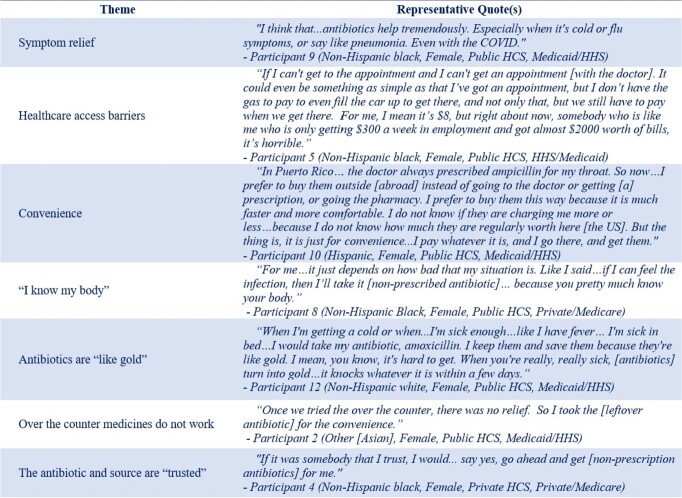
Representative participant quotes by theme

**Figure 1.**
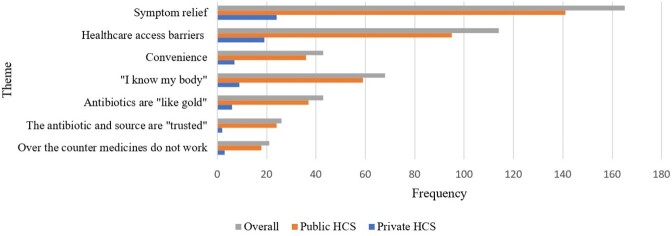
Overview of themes: Reasons to use nonprescription antibiotics by healthcare system (HCS)

**Conclusion:**

Qualitative analyses revealed the beliefs, practices, and healthcare system-related obstacles underlying nonprescription use. The concept of taking antibiotics for symptom relief (including for viral infections and pain) gives antibiotic stewardship programs a new direction with patient education. In addition, promoting more accessible primary care by reducing healthcare-access barriers may reduce patients’ usage of antibiotics outside of a healthcare setting.

**Disclosures:**

**Barbara Trautner, MD, PhD**, Genentech: Grant/Research Support|Peptilogics: Grant/Research Support

